# Koch’s “Lymph” in Lupus

**Published:** 1891-02

**Authors:** 


### Editorial.

### KOCH'S "LYMPH" IN LUPUS.

For the past two or three months we have been reading positive statements that *lupus vulgaris* was quickly cured by injections of this lymph. As the disease is noted for its recurrence in the scar even when several months have elapsed since an apparent cure, we were inclined to doubt the claims which were made. But it seems that it was not necessary to wait months in order to know the actual result. Credence, however, seems greater in this country than abroad. Our New York correspondent says in his letter of this issue that—"it may be regarded as proved that lupus can be at least temporarily cured in this way." We may take this as an expression of the view in New York, but other methods have "temporarily cured" the disease. In the January number of the (New York) *Journal of Cutaneous and Genito-Urinary Diseases* editorial mention is made of reported astonishing cures of long-standing cases, but the writer comments on the effects of the lymph, thus: "The test of time must of course determine whether or not such apparent cures will be permanent but from the reported action of the lymph on the tuberculous tissue and not on the micro-organisms one would be justified in anticipating relapses."

The *British Journal of Dermatology* for January goes farther in an editorial in which the action of the lymph is quite extensively considered. Quotes Dr. Thibierge of Paris as saying that he had not seen a *single case* of lupus in Berlin which was even *apparently cured* by the injections. Editor closes with the statement that not a single well authenticated case has had the disease "wiped out" and thoroughly removed.

